# A Monoclonal Antibody Produced in Glycoengineered Plants Potently Neutralizes Monkeypox Virus

**DOI:** 10.3390/vaccines11071179

**Published:** 2023-06-30

**Authors:** Adrian Esqueda, Haiyan Sun, James Bonner, Huafang Lai, Collin Jugler, Karen V. Kibler, Herta Steinkellner, Qiang Chen

**Affiliations:** 1 The Biodesign Institute, Arizona State University, Tempe, AZ 85287, USA; 2 School of Life Sciences, Arizona State University, Tempe, AZ 85287, USA; 3 Department of Applied Genetics and Cell Biology, University of Natural Resources and Life Sciences, 1180 Vienna, Austria

**Keywords:** monkeypox virus (MPXV), monoclonal antibody (mAb), plant-made antibody, plant-made pharmaceutical

## Abstract

The 2022 global outbreaks of monkeypox virus (MPXV) and increased human-to-human transmission calls for the urgent development of countermeasures to protect people who cannot benefit from vaccination. Here, we describe the development of glycovariants of 7D11, a neutralizing monoclonal IgG antibody (mAb) directed against the L1 transmembrane protein of the related vaccinia virus, in a plant-based system as a potential therapeutic against the current MPVX outbreak. Our results indicated that 7D11 mAb quickly accumulates to high levels within a week after gene introduction to plants. Plant-produced 7D11 mAb assembled correctly into the tetrameric IgG structure and can be easily purified to homogeneity. 7D11 mAb exhibited a largely homogeneous N-glycosylation profile, with or without plant-specific xylose and fucose residues, depending on the expression host, namely wild-type or glycoengineered plants. Plant-made 7D11 retained specific binding to its antigen and displayed a strong neutralization activity against MPXV, as least as potent as the reported activity against vaccinia virus. Our study highlights the utility of anti-L1 mAbs as MPXV therapeutics, and the use of glycoengineered plants to develop mAb glycovariants for potentially enhancing the efficacy of mAbs to combat ever-emerging/re-emerging viral diseases.

## 1. Introduction

Monkeypox virus (MPXV) is a zoonotic orthopoxvirus that causes a serious smallpox-like illness in humans with symptoms including fever, headache, and lymphadenopathy followed by intense rashes on the face and extremities [1]. MPXV was first identified as a pathogen in non-human primates and later recognized as a human pathogen [1]. Human cases of MPXV infection were originally restricted to the endemic regions in Central and West Africa, but since 2003 it has expanded to outside Africa and caused sporadic human infection in several non-African countries [2]. Since May of 2022, however, 86.516 confirmed cases have been reported in 110 countries outside the traditional MPXV endemic area [3]. Furthermore, most of these cases are not directly associated with traveling to endemic regions but mostly through human-to-human transmission [1]. The re-emergence of MPXV and its spread around the world indicates the potential of another large-scale epidemic such as the COVID-19 pandemic and calls for the development of control and preventative measures. While smallpox vaccines can provide cross-protection and new MPVX vaccines have been approved, monoclonal-antibody (mAb)-based prophylactics and therapeutics are still urgently needed for protecting the immunocompromised, elderly people, and individuals allergic to MPVX vaccines, as vaccines may not be able to induce a long and protective immune response or may cause unintended immunopathological effects in these subpopulations [1,4].

MPXV is an enveloped virus that has a genome consisting of double-stranded DNA. The MPXV genome shares high homology with the related vaccinia virus (VACV) and variola virus (VARV), the causative agent of smallpox, with a genome size of approximately 197 kb containing ~190 nonoverlapping open reading frames (ORFs) [5]. Studies with smallpox vaccines in animal models have shown that neutralizing antibodies are not only necessary but also sufficient for protecting animals from lethal dosages of infection by orthopoxvirus, including MPXV [6,7,8,9]. Some of the same studies, however, indicated that the activity of memory T cells was not essential for protectivity against VACV and MPXV in animal models [6,7]. The protectivity of the humoral immune response against smallpox in humans has also been demonstrated. The neutralizing epitopes of poxviruses have been studied extensively and many of them have been mapped to various viral proteins. However, the L1 protein has been found to be one of the most protective antigens among poxviruses [10]. The L1 protein is a membrane protein anchored on the surface of the poxviruses. This protein is found on the mature virion (MV) of poxviruses and is well conserved among orthopoxviruses, with its coding sequence being almost identical among MPVX, VARV, and VACV [11]. The L1 protein has also been shown to play a crucial role in viral entry into host cells [12,13]. Therefore, antibodies against the L1 protein may interrupt viral entry and confer the neutralization of MPXV and related poxviruses. Indeed, a mAb, 7D11, against a conformational epitope of VACV L1 protein was found to be potently neutralizing and to protect mice against lethal infection of VACV [11,14,15]. Therefore, anti-L1 mAbs such as 7D11 present a class of promising candidates for developing therapeutics against the re-emerging MPVX epidemics.

Protein-based biologics including mAbs are traditionally produced in platforms based on mammalian cell culture, with Chinese hamster ovary cell (CHO) being the most popular production host. In the last decade, plants have increasingly shown their importance as a platform in the industry of biologics. The utilities of plant-based platforms include both using plants as a production host for manufacturing biologics and as a system to develop novel biologics with new functionalities. For example, producing biologics in plants has been shown to be more economical and carry less risk of introducing human or animal pathogens [16]. Moreover, plants have been explored to develop biologics with superior potency or safety against various disease targets [17]. The contribution of plant-based platforms in biologic advancement and manufacturing can be best illustrated by the countermeasures developed for the current COVID-19 pandemic. Among the few vaccines licensed for SARS-CoV-2 prevention, a plant-produced vaccine has made its mark, as this virus-like particle-based vaccine does not require storage and transportation under ultra-cold conditions, and thereby, it has the potential to have a broader impact in parts of the world where a cold chain for biologics is not available [18]. Plants have also been shown to be one of the efficient systems in developing mAb-based therapeutics against SARS-CoV-2 infection. Plant-made mAbs have been shown to effectively neutralize various SARS-CoV-2 variants of concern (VOC) and block the initiation of the S-protein-mediated IL-6 pathway, which may lead to cytokine storm [19,20,21,22]. In addition, plants have also provided an efficient platform for rapidly identifying the optimal antibody isotypes and synergistic mAb cocktail partners for COVID-19 treatment [20]. These accomplishments are attributed to the robust nature of plant transient expression, which offers multiple advantages over the traditional systems based on stable transgenic plants. The superiority of the transient expression system includes production speed (within 2 weeks of gene delivery), high target protein accumulation levels, and versatility for quickly adapting the technology for new product development [16]. In addition, producing mAbs in plants may reduce production costs, as well as increase mAb potency and/or safety by manipulating antibody the Fc effector function via N-linked glycan modulation [23,24]. In addition to COVID-19, plant-made mAbs have been shown to be more efficacious and safer alternatives to their mammalian-made counterparts in preventing and treating various viral diseases [25,26,27].

In this study, we used wild-type (WT) and glycoengineered plants to develop a therapeutic mAb candidate (7D11) against MPXV infection. 7D11 mAb was expressed in *Nicotiana benthamiana* plants, reaching the highest level within 6–7 days of gene introduction. Mass-spectrometry (MS)-based glycan analysis of 7D11 mAb revealed largely homogeneous complex N-glycans, with and without plant-specific xylose and fucose, depending on the expression plant host line, namely WT or the glycosylation mutant ΔXF. 7D11 produced in plants assembled and folded correctly into the tetrameric IgG form. Functional activities of 7D11 mAb were revealed by specific recognition of its conformational epitope on MPXV-infected cells and a potent neutralizing activity against MPXV. Since 7D11 mAb produced in the glycoengineered plant line carried a homogenous N-glycoform that has been shown to enhance IgG effector functions, our results open the opportunity for future development of effective MPXV-neutralizing mAbs with enhanced efficacy through Fc effector function modulation.

## 2. Materials and Methods

### 2.1. Expression Vector Construction and Plant Expression

Variable region sequences for light chain (LC) and heavy chain (HC) of 7D11 mAb [11] were synthesized by GENEWIZ (South Plainfield, NJ, USA). Synthesized DNA fragments were genetically fused to a human kappa constant sequence of the LC and a human gamma IgG1 constant sequence of HC using restriction enzymes of EcoRI, NheI, and BamH1 [28]. BsaI, XbaI, and SacI were then used to clone LC and HC genes into a geminiviral-based plant expression vector named pBY11eK2Md, which was developed using bean yellow dwarf virus (BeYDV). The plant-expression vector was subsequently transformed into *Agrobacterium tumefaciens* strain EHA105. The *A. tumefaciens* strain with confirmed plant expression vector was then introduced into leaves of *N. benthamiana* plants via syringe agroinfiltration for transiently expressing 7D11 mAb as described previously [19]. *N. benthamiana* plants, a *Nicotiana* species with inherent advantages for transient gene expression (reviewed in [29]), were grown at 25 °C with a 16 h light/8 h dark cycle under 65% humidity [30].

### 2.2. Extraction and Purification of 7D11 mAb

7D11 mAb were extracted and purified according to published protocols [28,31]. Briefly, mAb-expressing *N. benthamiana* leaves for all plant lines were harvested 7 days post infiltration and homogenized in extraction buffer (1x phosphate-buffered saline (PBS) pH 5.2 with 10 mg/mL Na-L-ascorbate, 2 mM phenylmethylsulphonyl fluoride (PMSF), and 1 mM ethylenediaminetetraacetic acid (EDTA)). The pH of the homogenate was adjusted to 5.2 and was incubated at 4 °C overnight to precipitate host proteins. We then clarified the plant extract by spinning at 15,000× *g* at 4 °C for 30 min. The centrifugation was repeated two more times, followed by vacuum filtration using a 0.2-micron filter. The post-filtration extraction was defined as the clarified total soluble leaf protein extract, which was further processed by Protein-A (MabSelect, Cytiva, Marlborough, MA, USA)-based affinity chromatography to purify 7D11 mAb according to the manufacture’s protocol prior to further analysis.

### 2.3. SDS-PAGE and Western Blot Analysis

We performed SDS-PAGE and western blotting analysis based on our previously published protocols [20]. Briefly, mAbs were subjected to SDS-PAGE (4–20%) analysis under reducing and non-reducing conditions and were stained with Coomassie Blue R-250 to detect total protein content. Western blotting analysis was also performed by separating proteins either under reducing or non-reducing conditions using 4–20% acrylamide gels. The proteins on SDS-PAGE were then transferred to nylon-based membranes. Western blots were further processed by blocking the membranes with PBST (0.05% Tween-20 + 5% milk in 1x PBS), followed by incubating with a goat anti-human HC antibody (Southern Biotech, Birmingham, AL, USA) to detect 7D11 mAb HC. For detecting the LC of 7D11 mAb, an anti-human kappa chain antibody (Southern Biotech, Birmingham, AL, USA) was used. Both the anti-HC and anti-LC antibodies are conjugated with horseradish peroxidase (HRP). Membranes were then incubated with HRP substrate (Pierce West Pico Substrate for Western blotting, Thermo Scientific, Waltham, MA, USA) for 5 min. After washing, the membrane was detected in an ImageQuant instrument (Cytiva, Marlborough, MA, USA) to generate the western blot images.

### 2.4. ELISA

We employed a previously developed ELISA to quantitate the expression of 7D11 mAb in *N. benthamiana* leaves. This assay detects only the fully assembled 7D11 mAb with both HC and LC [20]. In brief, *N. benthamiana* leaves agroinfiltrated with 7D11 mAb LC and HC genes were harvested on 4–9 days post infiltration (DPI). Clarified total protein extract was obtained as described above, and the extract was serially diluted and transferred to 96-well plates in which the capture antibody (anti-human HC made in goat, concentration = 2 µg/mL, Southern Biotech, Birmingham, AL, USA) was immobilized, followed by blocking with 5% milk in 1x PBST. Dilutions of an Isotype IgG of known concentrations were used as a reference standard. The microtiter plates were washed 3 times with 1x PBST after one hour of incubation at 37 °C. The detection antibody (HRP-labelled anti-human kappa LC antibody, Southern Biotech, Birmingham, AL, USA) was then added to the plates and incubated for an hour, followed by washing with 1x PBST. After washing the plate, 7D11 mAb was detected by incubating with the HRP substrate (TMB, SeraCare Life Sciences Inc., Milford, CT, USA) for five minutes before adding the stop solution of 1 M H_2_SO_4_. The plates were then read in a spectrophotometer at 450 nm. The accumulation levels of 7D11 mAb in plant leaves (µg mAb/g leaf fresh weight (LFW)) were calculated with GraphPad Prism 9.0 (GraphPad, San Diego, CA, USA).

Indirect ELISA was used to investigate the specific binding of p7D11 mAb to the MPXV L1 protein (MedChemExpress, Monmouth Junction, NJ, USA) using a previously described method [32]. Briefly, 2 µg/mL of L1 was coated on 96-well plates overnight at 4 °C and blocked with 5% milk in 1x PBST. Serial dilutions of p7D11 mAb or a control isotype human IgG were made in 5% milk in 1x PBST. All wash steps between incubations were performed three times with 1x PBST. After blocking and washing the plates, dilutions of p7D11 along with an isotype IgG negative control were added to the plates and incubated for one hour at 37 °C. After washing, the plates were then incubated with HRP-conjugated goat anti-human IgG. The plates were then developed by incubating with TMB substrate for five minutes before adding the stope solution of 1 M H_2_SO_4_. Absorbance was read at 450 nm, and GraphPad Prism 9.0 was used to generate graphs and calculate approximate dissociation constants (KD) of p7D11 with the one-site specific-binding model.

### 2.5. Glycan Analysis

We characterized the N-glycosylation population of 7D11 mAb using mass spectrometry with our previously published procedures [23,32]. Briefly, 7D11 mAb was separated on SDS-PAGE, and HC was excised and digested with trypsin to generate glycopeptides, which were further identified by a liquid chromatography–electrospray ionization mass spectrometry (LC-ESI-MS) system (Orbitrap Exploris 480, Thermo Scientific, Waltham, MA, USA). Peaks representing Glycopeptides, consisting of various peptides and the associated N-glycan moieties, were identified by using FreeStyle 1.8 (Thermo Scientific, Waltham, MA, USA) with the manual glycopeptide searches option and Extract function for deconvolution. The molar ratios of the glycoforms are approximated by the height of peaks. The nomenclature defined by the Consortium for Functional Glycomics was used to annotate various N-glycans [33].

### 2.6. Immunofluorescence Staining

Vero E6 cells (obtained from ATCC #CCL81, Manassas, VA, USA) were plated at the density of 25,000 cells/well in a 96-well microtiter plate with a clear flat bottom. The medium used for such purpose is Dulbecco’s Modified Eagle’s Medium (100 μL, DMEM, Gibco, Waltham, MA, USA), which is supplemented with fetal bovine serum (FBS, Gibco, Waltham, MA, USA) at the final concentration of 10%. The cells were infected 24 h later with MPXV (WRAIR 7-61, BEI# NR-27) with a multiplicity of infection (MOI) of 0.9 and incubated for 24 h. Paraformaldehyde (4%) was then used to fix the cells. Subsequently, the cells were treated with 0.1% saponin (in phosphate-buffered saline) for permeabilization. Permeabilized cells were then incubated with 5 µg/mL of 7D11 mAb at 4 °C overnight. Cells were washed three times with 1x PBS with 0.1% Tween-20 and stained with an Alexa-488-conjugated goat anti-human light chain antibody from Southern Biotech (Birmingham, AL, USA). Images were taken using an Evos cell imaging system (ThermoFisher, Waltham, MA, USA).

### 2.7. MPXV Neutralization Assay

We used a focus-forming assay (FFA) to determine the neutralization potency of 7D11 mAb based on our established protocols [20]. Essentially, Vero E6 cells (25,000 per well, 100 µL) were plated in tissue culture plates (flat-bottom, 96-well) 24 h before the assay in DMEM, which is supplemented with FBS to the final concentration of 10%. 7D11 mAb was serially diluted in a 96-well microtiter plate (round bottom) using DMEM supplemented with FBS (2%). In the meantime, 2000 plaque-forming units (PFU) of MPXV were prepared by diluting the viral stock with DMEM supplemented with 2% FBS. 7D11 mAb dilutions were then mixed with the MPXV preparation and incubated at 37 °C for one hour. The mAb-7D11 mAb mixture was then added to the plated Vero E6 cells with a MOI of 0.9 and incubated at 37 °C for another additional hour. After incubation, a 100 µL/well overlay of MEM/methylcellulose was transferred to each well to cover the cells. The cells were then incubated at 37 °C for 24 h. After incubation, cells were processed sequentially by removing the overlay and being treated with paraformaldehyde (4%) for fixation, followed by permeabilization with saponin (0.1%) and washing with bovine serum albumin (BSA in 1x PBS) six times. For the staining of the infected cells, cells were incubated with a rabbit anti-E3L antibody with a dilution of 1:1000 dilution, followed by incubating with a goat anti-rabbit IgG that was conjugated to HRP (Sigma, St Louis, MO, USA). Finally, KPL TrueBlue substrate (SeraCare Life Sciences Inc., Milford, CT, USA) was added to each well and incubated for 15 min at room temperature. The plate was then washed with deionized water and imaged, and foci were quantified using an AID Spot Reader (Strassberg, Germany). GraphPad Prism 9.0 was used to analyze the FFA data. The following formula was used to calculate the neutralization percentage (average total foci number in wells not treated with antibody–average total foci number in wells treated with antibody)/average total foci number in wells not treated with antibody. Experiments were repeated at least two times independently and triplicate samples were used for each concentration of the antibody.

### 2.8. Statistical Analyses

GraphPad Prism software version 9.0 was used to perform statistical analyses. Specifically, a Normality test (D’Agostino & Pearson test) was performed to check the distribution of data. In all cases, normal (Gaussian) distribution was confirmed for data of 7D11 mAb expression, binding to L1 antigen, and neutralization. Once confirmed, one-way ANOVA was used to compare the expression levels of 7D11 mAb on different days post agroinfiltration and to compare specific binding of MPXV L1 protein by plant-produced 7D11 mAb and the isotype IgG control. *T*-tests were used to compare the neutralization activity of 7D11 mAb produced in different plant lines. A *p* value of <0.05 indicated a statistically significant difference.

## 3. Results

### 3.1. Expression of 7D11 Monoclonal Antibody in Nicotiana Benthamiana

**Figure 1:**
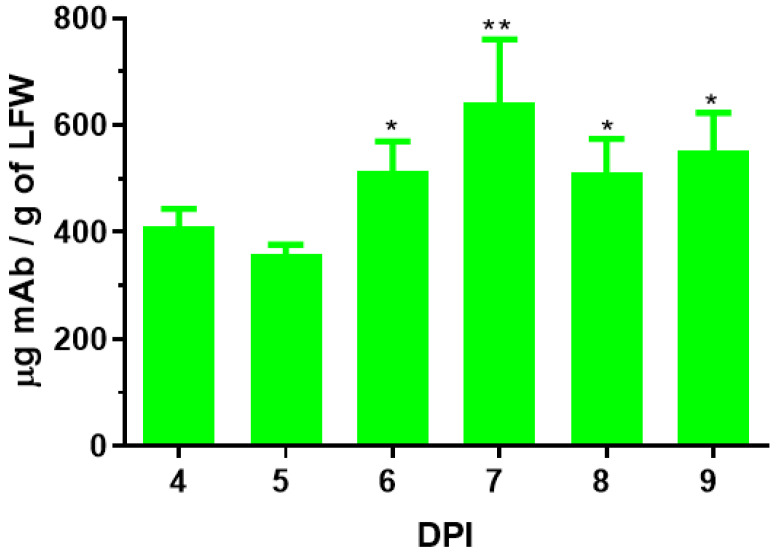
Expression time course of 7D11 mAb in leaves of *N. benthamiana*. Total proteins were extracted from leaves of *N. benthamiana* plants that were agroinfiltrated with constructs coding for 7D11 mAb light chain and heavy chain on 4–9 days post infiltration (DPI). The accumulation levels of p7D11 were measured by an ELISA that solely quantitated IgG molecules with assembled LC and HC. Data (mean ± SD) presented are from two independent infiltration experiments. Each data point was performed with technical triplicates. ** and * indicate *p* values < 0.0064 and <0.0193 (compared with 4 and 5 DPI), respectively, from one-way ANOVA analysis.

**Figure 2:**
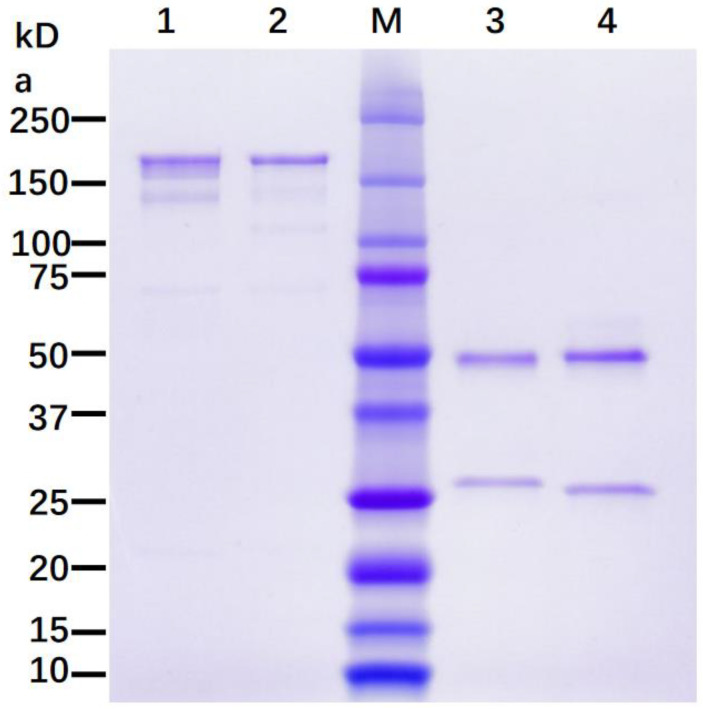
Assembly and purification of plant-made 7D11 mAb. 7D11 mAb was isolated from infiltrated leaves of ΔXF *N. benthamiana* plants followed by purification via Protein-A-based affinity chromatography. Purified p7D11 mAb was separated on SDS-PAGE along with an IgG isotype control. SDS-PAGE was performed either without using reducing reagent (Lanes 1 and 2) or with reducing reagent (Lanes 3 and 4), followed by staining with Coomassie blue. Lanes 1 and 4: IgG isotype reference. Lanes 2 and 3: p7D11 mAb. One representative result from multiple independent experiments is presented.

**Figure 3:**
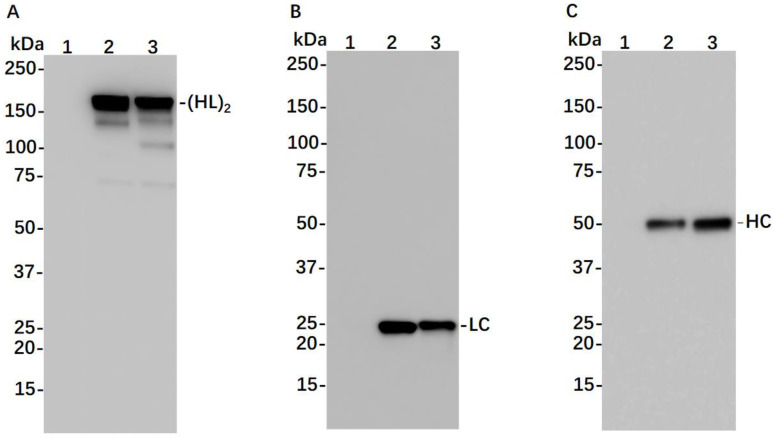
Western blotting analysis of 7D11 mAb derived from *N. benthamiana*. Total leaf proteins were extracted from ΔXF *N. benthamiana* leaf blades infiltrated either with 7D11 construct or buffer and were separated on SDS-PAGE along with an IgG isotype control under non-reducing (**A**) or reducing (**B**,**C**) conditions. Separated proteins were electro-transferred to nylon-based membranes and detected with antibodies against human kappa LC (**A**,**B**) or human gamma HC (**C**). Lane 1: total protein extracted from buffer-infiltrated leaves. Lane 2: IgG isotype control. Lanes 3: proteins isolated from leaves infiltrated with 7D11 construct. One representative blot from multiple experiments is shown.

To transiently express 7D11 mAb in plants, the genes for the LC and HC of this mAb were cloned in a plant-expression vector based on BeYDV, and the 7D11 mAb gene construct–carry vector was agroinfiltrated into leaves of WT or in ΔXF *N. benthamiana* plants, a glycosylation mutant that lacks plant-specific xylose and fucose residues on N-glycans [23]. Using an ELISA that detects only the fully assembled form of IgG, 7D11 mAb was detected as early as 4 days post infiltration (DPI) of the transgene. The accumulation levels of 7D11 mAb at 6–9 DPI were higher than those of 4–5 DPI (*p* < 0.0064 for 7 DPI vs. 4–5 DPI; *p* < 0.0193 for 6, 8, and 9 DPI vs. 4–5 DPI) with a peak expression average level of 642 µg of mAb per gram of LFW (Figure 1). Using Protein-A-based affinity chromatography, plant-produced 7D11 mAb (p7D11 mAb) was purified to homogeneity in a degree (~95%) similar to that of Chinese hamster ovary (CHO) cell-produced mAb purified by the same process (Figure 2). Furthermore, western blot analysis confirmed that p7D11 mAb was assembled to the expected heterotetrameric IgG form (Figure 3A) containing LC (Figure 3B) and HC with the expected molecular mass (Figure 3C).

### 3.2. N-Linked Glycosylation Analysis of p7D11 mAb

The N-glycosylation of 7D11 mAb produced in the two different plant lines was analyzed by LC-ESI-MS, as the composition of the glycans can impact an antibody’s Fc domain-mediated effector function [34]. p7D11 mAb expressed in WT plants (p7D11^WT^ mAb) was found to carry complex-type N-glycans with N-acetylglucosamine (GlcNAc) as the terminal glycan residues at least on one of the two termini, and all glycans were decorated with plant-specific core xylose and 1,3-linked fucose (GnGnXF_3_/GnGnX; GnMXF_3_/MMXF_3_, Table 1, Figure S1A). This result is consistent with the N-glycan profiles of other mAbs produced in WT *N. benthamiana* plants [23]. In contrast, p7D11 mAb produced in ∆XF plants (p7D11^∆XF^ mAb) exhibited exclusively a complex N-glycosylation pattern with GlcNAc residues as terminal glycans but lacking the plant-specific xylose and fucose (Table 1, Figure S1B). Notably, p7D11^∆XF^ mAb displayed GnGn, a mammalian-type N-glycosylation, as its predominant glycoform with 93% homogeneity (Table 1). Besides the abovementioned glycosylated p7D11 mAbs, ~20% of non-glycosylated Fc was detected for p7D11^WT^ mAb and p7D11^∆XF^ mAb (Figure S1).

p7D11 mAb heavy chain was extracted from SDS-PAGE, which was then digested with trypsin, followed by LC-ESI-MS analysis. FreeStyle 1.8 software was used to identify peaks of glycopeptide and to assign the percentage to each peak by approximating the molar ratios of each peak height. The annotation is according to the convention proposed by the Consortium for Functional Glycomics. Further details are in Figure S1.

### 3.3. p7D11 mAb Recognizes Viral Antigen in Monkeypox Virus-Infected Vero Cells

**Figure 4:**
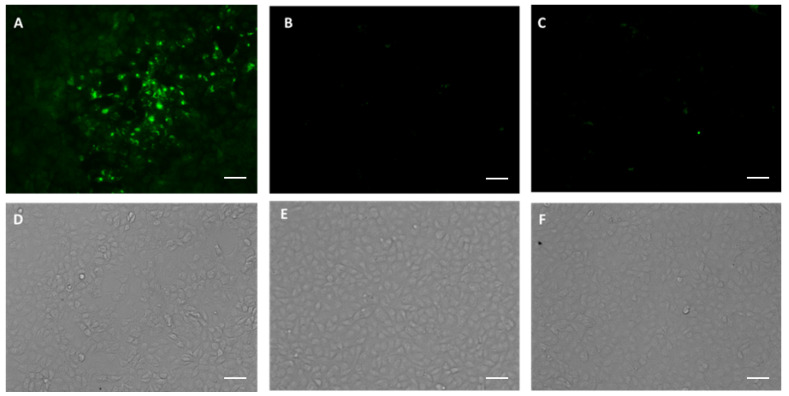
p7D11 mAb recognition of viral antigen in MPXV-infected cells by immunofluorescence microscopy. Vero E6 cells were plated in a clear-bottom microtiter plate and infected with MPXV at an MOI of 0.9 for 24 h. Cells were then fixed and permeabilized with 4% paraformaldehyde and 0.1% saponin, respectively. Permeabilized cells were incubated with p7D11^∆XF^ mAb, followed by staining with an anti-human Kappa LC antibody conjugated to Alexa 488 (**A**,**D**). Uninfected Vero E6 cells were processed and stained with p7D11^∆XF^ mAb and Alexa-488-conjugated goat anti-human Kappa antibody in parallel (**B**,**E**) as a negative control. MPXV-infected cells that were stained directly with Alexa-488-conjugated goat anti-human Kappa antibody (**C**,**F**) serve as an additional negative control (secondary-antibody-only control). Stained cells were photographed with an Evos imaging system using an Alexa 488 filter for immuno-stained MPXV L1 protein (**A**–**C**) or with a bright field setting (**D**–**F**). Scale bar shown in the images is 50 µm.

**Figure 5:**
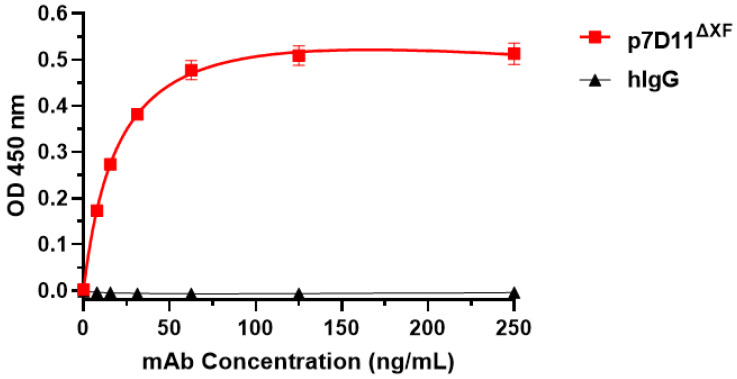
Specific binding of p7D11 mAb to MPXV L1 protein. Serial dilutions of p7D11 mAb or an isotype human IgG (hIgG) control were incubated with MPXV L1 protein that was immobilized on ELISA plates. Specific binding of p7D11 mAb to L1 was detected with HRP-conjugated goat anti-human IgG. The absorbance at 450 nm (Mean ± SD) from at least two independent experiments with technical triplicates was plotted, and KD was calculated with GraphPad Prism 9.0.

We next investigated the functional characteristics of p7D11^∆XF^ by testing if the mAb can bind to the target viral antigen by immunofluorescence staining. Indeed, sequential staining of MPXV-infected Vero cells with p7D11^∆XF^ mAb and an Alexa-488-conjugated secondary antibody (goat anti-human IgG) showed specific binding of p7D11^∆XF^ mAb to an antigen in infected cells (Figure 4A). In contrast, no specific antigen binding was detected with p7D11^∆XF^ mAb in uninfected Vero cells (Figure 4B), indicating the stained antigen is MPXV-specific. Likewise, staining of infected Vero cells directly with the Alexa-488-conjugated secondary antibody did not show any specific binding (Figure 4C). ELISA analysis confirmed the specific binding of p7D11^∆XF^ mAb to MPXV L1 protein in a dose-dependent manner (Figure 5), with a dissociation constant (KD) of 0.1 nM, suggesting a relatively high binding affinity for MPXV L1. p7D11^WT^ mAb also exhibited an L1-binding curve almost identical to that of p7D11^∆XF^ (*p* = 0.95 by one-way ANOVA) (Figure S2). In comparison, the isotype control IgG did not show binding activity to L1 protein (*p* = 0.0002 compared with p7D11^∆XF^) (Figure 5). Together, these results show plant-produced 7D11 mAb specifically recognized the MPXV L1R antigen.

### 3.4. Plant-Produced 7D11 mAb Potently Neutralizes Monkeypox Virus

**Figure 6:**
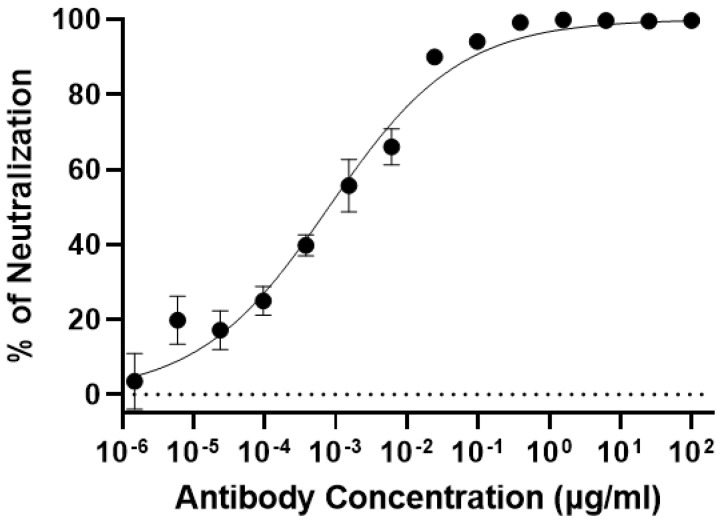
Neutralization of Monkeypox virus by p7D11 mAb. p7D11^∆XF^ mAb was serially diluted and mixed with MPXV before adding to Vero E6 cells. After 24 h of incubation, cells were processed by fixation and permeabilization, and then stained for MPXV foci using an antibody against E3L antibody and an HRP-conjugated secondary antibody. Counted Foci were used to generate neutralization curves, calculate the percentage of neutralization, and determine IC_50_ with GraphPad Prism 9.0. At least two independent experiments were performed with technical triplicates for the presented results (mean ± SD).

After confirming the specific binding of p7D11^∆XF^ mAb to the target antigen, the neutralizing potency of the mAb was determined by a foci-forming assay (FFA) using the authentic MPXV. Our result showed that p7D11^∆XF^ mAb potently neutralized live MPXV (Figure S3). Specifically, the half-maximal inhibitory concentration (IC_50_) of p7D11^∆XF^ mAb against MPXV was 0.78 ng/mL (Figure 6). p7D11^WT^ mAb also showed a neutralization pattern similar to that of p7D11^∆XF^ (*p* = 0.32) (Figure S4). Compared with the reported activity (IC_50_ = 10 ng/mL) of mammalian cell-produced 7D11 mAb against VACV [14], our result indicated that p7D11 mAb has a comparable neutralizing activity against MPXV.

## 4. Discussion

The 2022 worldwide outbreaks of MPXV and increased human-to-human transmission urge the development of prophylactics and therapeutics to prevent the further spread of this virus and to protect and treat people who are allergic to current MPXV vaccines or unable to develop a protective response from vaccination. We choose 7D11 mAb for the evaluation as a potential therapeutic because previous studies have demonstrated its high neutralizing activity against VACV, and it recognizes an epitope that is well conserved among poxviruses [11]. Our results show the rapid expression of 7D11 in *N. benthamiana* plants with a peak accumulation level at 642 µg/gram LFW 6–9 days posts DNA construct delivery. Compared with other mAbs that were recently developed by our group using the same plant-expression vector [19,32], the accumulation level of p7D11 mAb in leaves is much higher, indicating its potential for potential large-scale production. Published reports on the expression levels of 7D11 mAb in non-plant-based systems are currently not available. However, we can speculate that its expression levels in mammalian cell culture based systems would be higher than what we reported here. Nevertheless, the yield of p7D11 mAb can be further increased by co-expressing chaperons [35] or by employing improved expression vectors [36] to increase the feasibility for commercial production. We also showed that p7D11 mAb assembled correctly into the tetrameric structure of IgG without any apparent degradation or truncation. The proper folding of p7D11^∆XF^ mAb was confirmed by the specific binding to its antigen on MPVX-infected Vero cells. The ELISA analysis with purified MPXV L1 antigen yielded a dissociation constant of 0.1 nM, indicating a relatively high binding affinity of p7D11 mAb for MPXV L1. Of note, plant-produced 7D11^∆XF^ mAb exhibited a strong neutralization potency against live MPXV with an IC_50_ value of 0.78 ng/mL, at least as potent as the reported activity (10 ng/mL) against VACV [14]. Since it has been reported that administration of 7D11 mAb via intraperitoneal injection 1 day before challenge protected Balb/c mice from lethal infection of VACV [37], the superior neutralizing potency against MPXV suggests that p7D11^∆XF^ mAb has the potential to be used as prophylactics/therapeutics in preventing/treating MPXV infection. As animal models for in vivo study of vaccines and therapeutics are being rapidly developed/optimized [38], further testing of the efficacy of 7D11 mAb or 7D11-containing mAb cocktails in these models may shed new light on the molecular mechanism(s) of the protectivity of these mAbs against MPXV.

While plant-based systems still face major technical and regulatory challenges in improving mAb yield and in obtaining U.S. Food and Drug Administration approval for plant-made mAbs, the production of 7D11 mAb in *N. benthamiana* may provide the opportunity to develop therapeutics that have superior efficacy and/or are more economical against the re-emerging MPXV epidemics. For example, plant-based production platforms allow scalable mAb production using inexpensive facilities and economical upstream processes [24]. Techno-economic analyses have shown that mAb production based on transient expression in *N. benthamiana* plants can result in a >50% reduction in the production cost of goods compared with the traditional mammalian cell culture based production platforms at similar manufacturing scales [16]. This advantage is particularly important for the development and manufacturing of mAb-based therapeutics against MPXV infection and other emerging and re-emerging diseases, as the affordability of countermeasures is a major factor in protecting people in the developing world. The facile linear scalability of the plant-based platform may also increase the feasibility of providing the large amount of mAbs that may be demanded by the sheer scale of the potential global outbreaks. The unique biology of plants also allows plant-produced mAbs to exhibit a homogenous N-glycosylation profile to a degree that cannot be achieved currently in mammalian-cell-based systems [23]. Indeed, MS analysis indicated that p7D11 mAb expressed in ΔXF plants exhibited a single dominant homogenous glycan form, namely human-like GnGn structures. This ensures the compliance of p7D11^∆XF^ mAb to the requirements from regulatory agencies for product homogeneity and consistency. This also addresses its compliance with policies regarding the elimination of non-mammalian glycans in human therapeutics to minimize immunogenicity [23]. It should be pointed out that all results from human clinical trials of plant-made biologics to date have shown that they are not particularly immunogenic, and even the presence of plant-specific glycans does not induce any unwanted side effects [39,40].

More importantly, the N-glycosylation pattern, as generated herein by p7D11^∆XF^ mAb, may significantly enhance the mAb’s activity, as shown for other fucose-free anti-viral mAbs [41]. Fc effector functions allow antibodies to clear pathogens via complement or immune-cell-mediated mechanisms independent of neutralization [42,43]. For example, previous research has demonstrated that in addition to neutralization, antibody Fc-mediated effector functions via complement and various Fc receptors are crucial mechanisms of protection in VACV challenge studies using mouse models [44]. Since p7D11 mAbs carry an afucosylated GnGn glycoform that has been shown to have enhanced complement-dependent cytotoxicity (CDC) and antibody-dependent cellular cytotoxicity (ADCC) activity by increasing binding to FcγRs [45,46], p7D11^∆XF^ mAb may have other mechanisms of viral elimination in vivo in addition to neutralizing. This supports the hypothesis that compared with mammalian cell-produced 7D11 mAb, the in vivo efficacy of p7D11^∆XF^ mAb may have been increased via Fc effector functions. These warrant future comparative in vitro and in vivo studies for the CDC and ADCC activity of 7D11 mAb, where the inclusion of 7D11 mAb glycovariants produced by various non-plant-based systems is absolutely essential. Notably, about 20% of p7D11 mAb variants are non-glycosylated. This reveals the issue of underglycosylation that is occasionally observed on plant-produced IgGs [23]. Glycosylation efficiency may be enhanced by the co-expression of oligosaccharyltransferases, enzymes that transfer a preassembled oligosaccharide to polypeptides in the endoplasmic reticulum [23].

To our knowledge, this is the first report of developing mAb-based therapeutics in plants for the prevention/treatment of MPXV. While the results are promising, further analyses of viral elimination by alternative mechanisms such as CDC and ADCC and efficacy testing in relevant animal models are needed to demonstrate the full potential of plant-made 7D11 mAb. As MPXV outbreaks continue to reemerge, there is a need to monitor the emergence of mutants and quickly develop therapeutic mAbs that maintain neutralization and in vivo efficacy. We suggest that this can be most efficiently accomplished by developing mAbs with potent effector functions as well as by using an expression platform such as plant-based systems that can quickly screen for mAb cocktails with synergistic interactions. In summary, this study suggests the potential of anti-L1 mAbs as potent MPXV neutralizing therapeutics, as well as the utility of platforms based on plant transient expression in contributing to biologic development against the ever-emerging/re-emerging viral outbreaks.

## Figures and Tables

**Table 1:** N-Glycan Profile of 7D11 mAb derived from WT and ΔXFT *N. benthamiana* leaves.

Major N-Glycan Species	Schematic Presentation	p7D11^ΔXF^ (%)	p7D11^WT^ (%)
**GnGn**	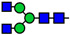	**93**	**10**
**GnGnX/GnGnXF_3_**	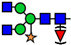		**68**
**MMXF_3_/GnMXF_3_**	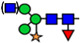		**22**
**MGn**	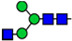	**7**	
** 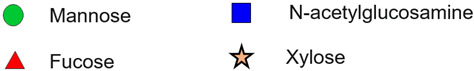 **			

## Data Availability

The data presented in this study are contained within this article.

## Supplementary Materials

The following supporting information can be downloaded at https://www.mdpi.com/article/10.3390/vaccines11071179/s1. Figure S1: MS spectra of 7D11 mAb produced in ΔXFT and WT N. *benthamiana plants*; Figure S2: Specific binding of p7D11^WT^ mAb to MPXV L1 protein; Figure S3: Focus-forming assay (FFA) to determining the neutralization potency of p7D11 mAb against MPXV; Figure S4: Neutralization of Monkeypox virus by p7D11^WT^ mAb.
