# Supplementary material for: A Monoclonal Antibody Produced in Glycoengineered Plants Potently Neutralizes Monkeypox Virus
Source: Vaccines (Basel). 2023 Jun 30;11(7):1179. doi: 10.3390/vaccines11071179 (PMC10416152; doi:10.3390/vaccines11071179)
Supplement: Supplementary file 1 [file vaccines-11-01179-s001.zip › vaccines-2390924-supplementary.pdf]

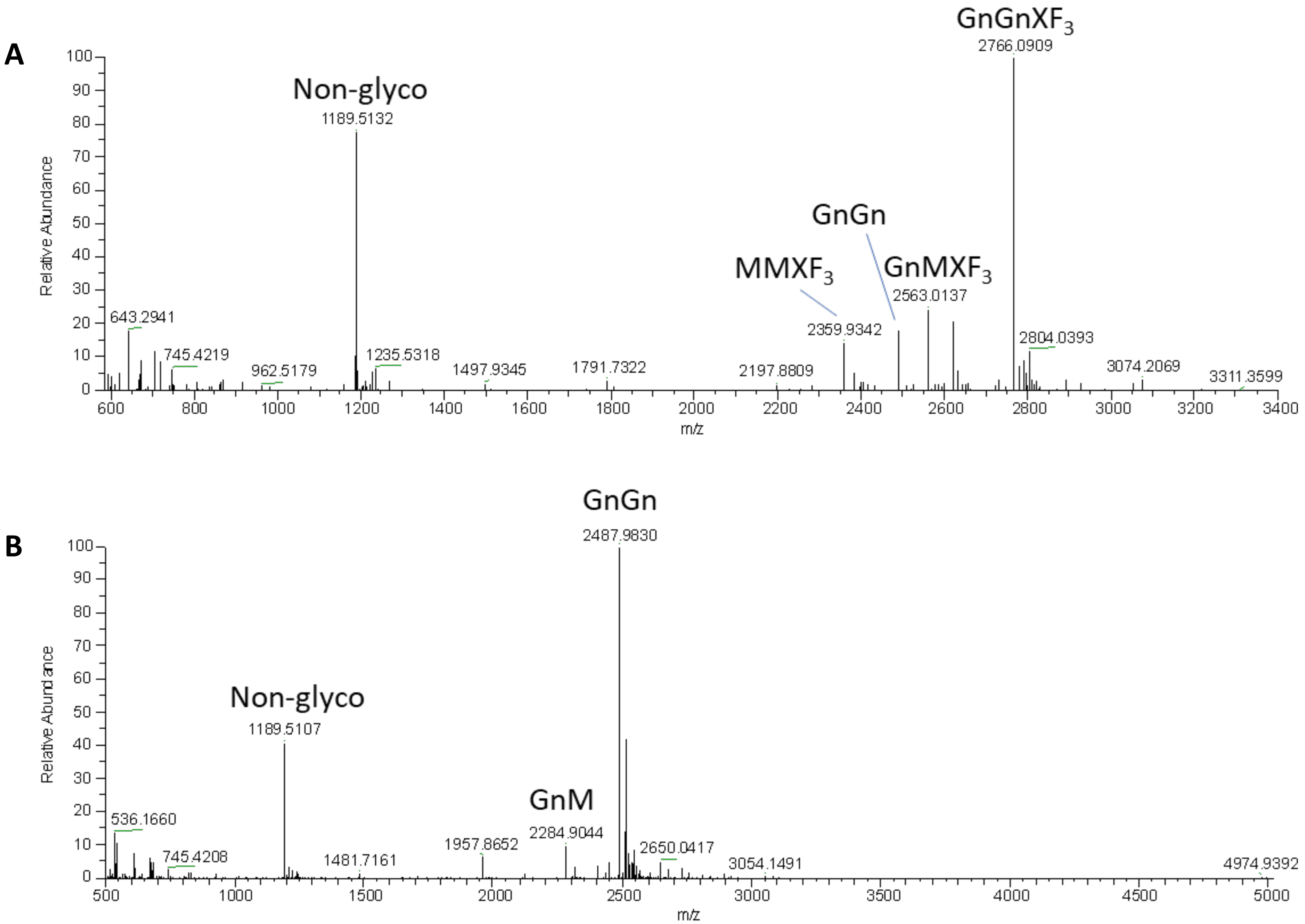

Figure S1. MS spectra of 7D11 mAb produced in ΔXFT and WT *N. benthamiana* plants

Heavy chain of p11D7^{WT} (A) or p7D11$^{\Delta XF}$ (B) was extracted from SDS-PAGE, trypsin digested and analyzed by LC-ESI-MS. Peaks of glycopeptide were identified using FreeStyle 1.8. Glycan annotation is based on the nomenclature of Consortium for Functional Glycomics.

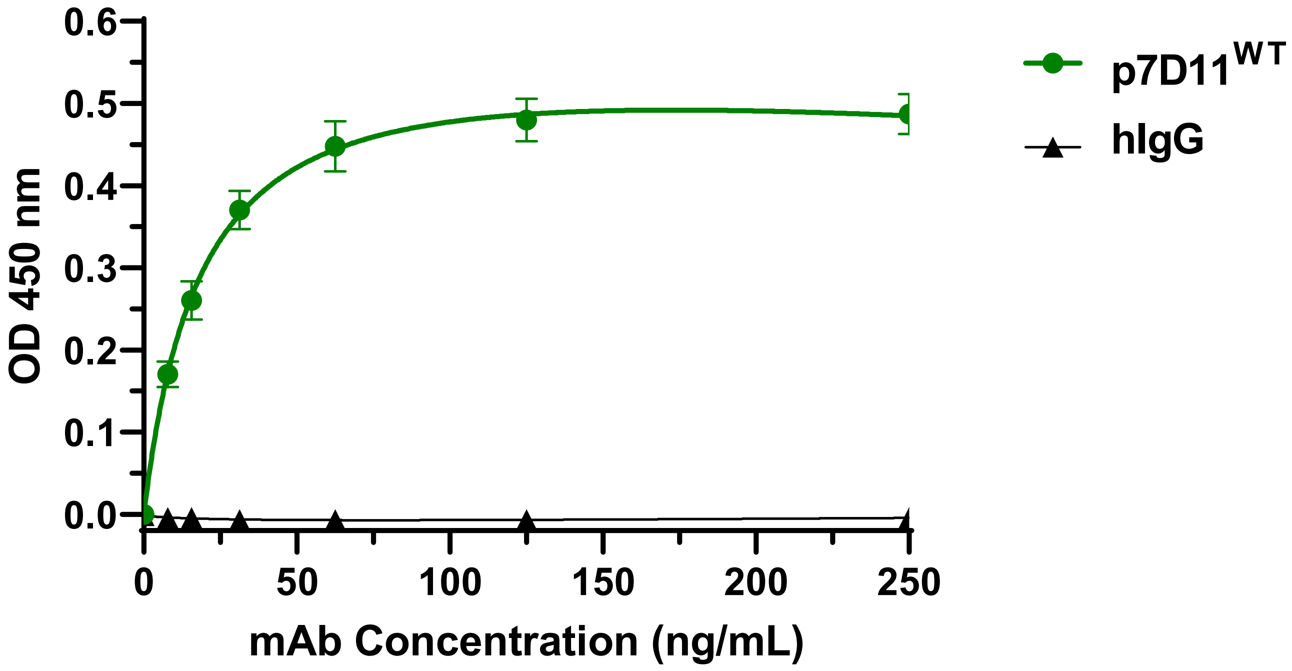

Figure S2: Specific binding of p7D11^{WT} mAb to MPXV L1 protein.

MPXV L1 protein that was immobilized on ELISA plates and incubated with dilutions of p7D11^{WT} mAb or an isotype human IgG (hIgG) control. Binding of p7D11 mAb to L1 was detected with HRP-conjugated goat anti-human IgG. At least two independent experiments with technical triplicates was performed to calculate the KD with GraphPad Prism 9.0.

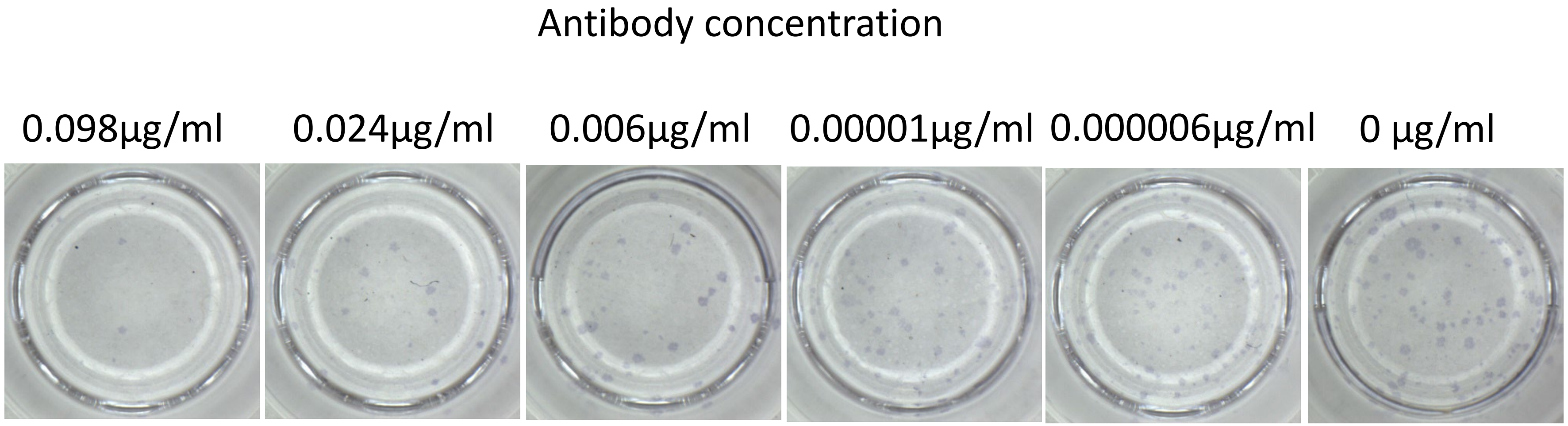

Figure S3. Focus-forming assay (FFA) to determining the neutralization potency of p7D11 mAb against MPXV.

Serially diluted p7D11$^{\Delta XF}$ mAb was mixed with MPXV and then added to Vero E6 cells that were plated in tissue culture plates. After 24 hours of incubation, cells were processed by fixation and permeabilization, and then stained for MPXV foci using an antibody against E3L antibody and an HRP-conjugated secondary antibody. Foci were photographed and counted to generate neutralization curves. Representative results from at least two independent experiments with technical triplicates were shown.

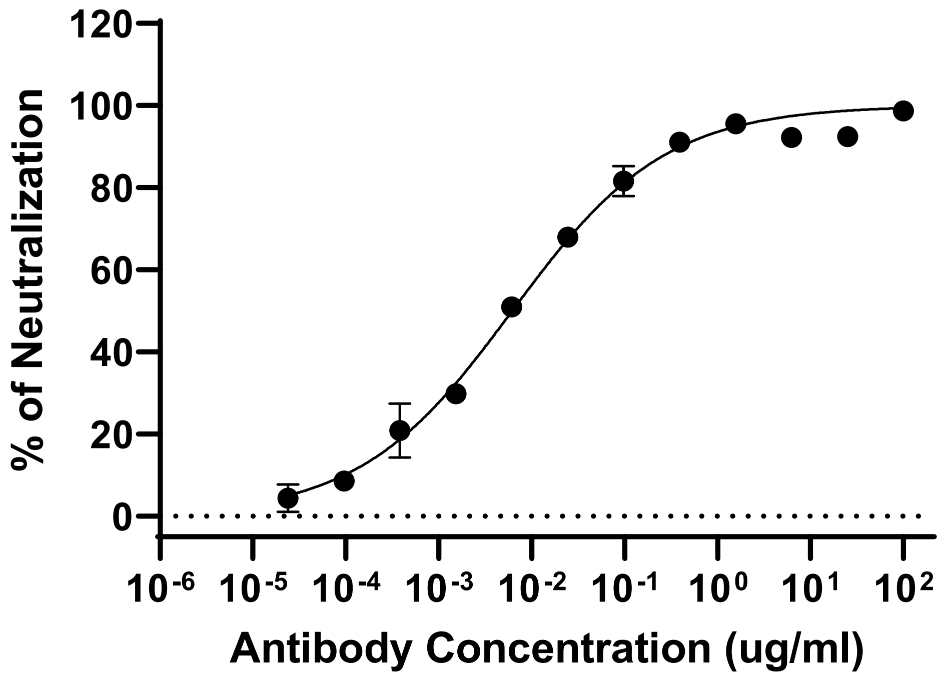

Figure S4: Neutralization of Monkeypox virus by p7D11^{WT} mAb.

p7D11^{WT}mAb was serially diluted and mixed with MPXV before incubating with Vero E6 cells for24 hours. After incubation, cells were fixed, permeabilized, and stained with a rabbit anti-E3L antibody followed by an HRP-conjugated goat anti-rabbit IgG. Foci were counted to determine the percent neutralization and IC_{50} calculated using GraphPad Prism 9.0. At least two independent experiments performed in technical triplicates.
